# Definitive radiotherapy for local and metastatic lesions in prostate cancer patients with oligometastases

**DOI:** 10.3389/fonc.2025.1662567

**Published:** 2025-11-03

**Authors:** Bichun Xu, Xianzhi Zhao, Yiyin Liang, Weiwei Zhang, Liang Chen, Yusheng Ye, Jie He, Jiaojiao Tong, Yangyang Gong, Judong Luo, Huojun Zhang

**Affiliations:** ^1^ Department of Radiation Oncology, The First Affiliated Hospital of Naval Medical University, Shanghai, China; ^2^ Department of Radiotherapy, Tongji Hospital, School of Medicine, Tongji University, Shanghai, China; ^3^ Department of Radiotherapy and Oncology, The Second Affiliated Hospital of Soochow University, Suzhou, China

**Keywords:** radiotherapy, prostate cancer, oligometastases, definitive, overall survival

## Abstract

**Background:**

Few studies explore the role of definitive radiotherapy for prostate and all metastases in the treatment of low-burden oligometastatic prostate cancer (omPCa). This study aimed to investigate the potential survival benefit of this approach. Moreover, it is the first study to report the outcomes of definitive radiotherapy for local and distant metastatic lesions in patients with omPCa from China.

**Methods:**

A retrospective analysis was conducted on patients with omPCa who received definitive radiotherapy for the primary site and metastatic lesions between July 2012 and June 2022. The inclusion criteria mandated fewer than 5 oligometastases, excluding regional lymph nodes by imaging examinations with no prior radiotherapy or radical prostatectomy for omPCa. Overall survival (OS) was the primary endpoint, and biochemical progression-free survival (bPFS) and radiological progression-free survival (rPFS) were the secondary endpoint. The Kaplan-Meier method was used to estimate survival rates. Univariate and multivariate analyses were conducted using Cox proportional hazards regression models.

**Results:**

A total of 33 patients, including 31 *de novo* oligometastatic hormone-sensitive prostate cancer (omHSPC) patients and 2 oligometastatic castration-resistant prostate cancer (omCRPC) patients, were enrolled in the study. The median follow-up was 38.8 months (range: 4.2–70.6 months). The OS rates of 2-, 3-, and 5-year after treatment were 100.0%, 95.7%,and 81.2%, respectively. Factors correlating with poorer survival were pre-radiotherapy CRPC status, symptomatic lesions, and prior transurethral resection of the prostate (TURP). Multivariate analysis revealed potential associations: concomitant androgen deprivation therapy (ADT) or chemotherapy, non-CRPC status pre-radiotherapy. Lymph node and bone metastases together increased the risk of biochemical recurrence. Acute adverse reactions of Grade 3+ were absent; chronic Grade 3 reactions were 3.0%.

**Conclusion:**

Definitive radiotherapy for local/metastatic lesions demonstrates promising survival with manageable toxicity in omPCa.

## Background

Prostate cancer (PCa) represented the second most common cancer and the fifth leading cause of death among patients, with 1.4 million new cases and 375,000 deaths worldwide in 2020 (1). The majority of diagnoses relate to the use of screening prostate-specific antigen (PSA), whereas the lack of routine PSA screening in the Chinese population often leads to diagnosis at a more advanced stage compared to the populations in Europe and the United States (2).

PCa treatment is stage-dependent, shifting from curative local therapies like surgery or radiotherapy for localized disease to systemic management with androgen deprivation therapy (ADT) for metastatic disease. In recent years, the treatment paradigm for advanced prostate cancer has undergone a revolutionary shift, characterized by earlier treatment intensification, an emphasis on precision medicine, and the adoption of novel therapeutic modalities. In the metastatic hormone-sensitive prostate cancer (mHSPC) setting, “triplet therapy” comprising ADT, docetaxel, and a novel androgen receptor signaling inhibitor (ARSI) has been demonstrated to provide superior survival benefits for patients (3). For metastatic castration-resistant prostate cancer (mCRPC), the application of PARP inhibitors has been proven effective in patients with specific DNA damage repair gene mutations, ushering in an era of genome-guided therapy (4). Concurrently, PSMA-targeted radioligand therapy (^177^Lu-PSMA-617) has become a new standard of care for heavily pre-treated mCRPC patients (5), establishing the era of “theranostics.” These therapeutic advances have been paralleled by synchronous developments in diagnostic technology, as prostate-specific membrane antigen positron emission tomography-computed tomography(PSMA PET/CT) has been proven to be far superior to conventional imaging, enabling more precise disease staging and treatment planning (6).

The profound efficacy of modern systemic therapies in prolonging patient survival has directed significant clinical attention toward oligometastatic prostate cancer (omPCa), an intermediate clinical state between localized and widespread metastatic disease. While a universal consensus is still evolving, omPCa is commonly characterized by a limited number of metastatic lesions (e.g., ≤ 5), though precise definitions based on lesion quantity and location remain under investigation (7). According to the CHAARTED study, the metastatic disease can be classified as high volume (visceral metastases or four or more bone metastases, with at least one of the bone lesions located outside the spine or pelvis) or low volume (not high) (4, 8, 9). This conceptualization challenges the traditional treatment paradigm. Although systemic therapy remains the foundational treatment for metastatic prostate cancer (mPCa) (10, 11), there is an emerging therapeutic rationale that, for this select patient subgroup, aggressive cytoreductive local therapies targeting all visible disease may ultimately improve survival outcomes.

Although the role of local therapy for the primary tumor in mPCa has been historically controversial (12), systemic therapy has served as the traditional cornerstone. However, this treatment paradigm is undergoing a profound evolution with the emergence of high-level evidence. Two pivotal randomized controlled trials reported that radiotherapy to the primary significantly improved outcomes for patients with newly diagnosed low-metastatic-burden mPCa (13–15). In particular, the STAMPEDE trial (Arm H) demonstrated a significant OS benefit, a finding reinforced by its long-term follow-up results (15). More recently, the PEACE-1 trial further substantiated this benefit within the context of modern intensified systemic therapy (triplet therapy), confirming that prostate radiotherapy continues to improve radiographic progression-free survival (rPFS) and castration resistance-free survival even alongside potent androgen receptor signaling inhibitors and docetaxel (16). These findings have reshaped clinical practice, as reflected in the 2024 Advanced Prostate Cancer Consensus Conference (APCCC) report, where a strong consensus now supports prostate-directed radiotherapy for *de novo* low-volume mHSPC patients (17). Advanced radiotherapy, including brachytherapy (BRT) and stereotactic body radiation therapy (SBRT), is a compelling form of local consolidative treatment (LCT) over radical prostatectomy (RP) due to its favorable impact on quality of life (QoL). However, the survival benefit of comprehensive radiotherapy targeting both the primary tumor and all oligometastatic lesions in patients with omPCa remains unproven.

The objective of this retrospective study was to investigate the potential survival benefit of definitive radiotherapy in patients with omPCa by analyzing patients from a single center who received radiotherapy to all lesions, including the primary site and metastatic lesions.

## Materials and methods

### Patient characteristics

All procedures were performed in accordance with relevant guidelines, and regulations and the study was approved by our institutional review board. Informed consent was obtained from all participants. The research was further approved by the Ethics Committee of the First Affiliated Hospital of Naval Medical University. Prior to conducting this study, oncologists performed a retrospective analysis of all patients to assess their eligibility for inclusion. Specifically, we conducted a retrospective review of patients with omPCa who received treatment at our institution from July 2012 to June 2022. The inclusion criteria for patients were as follows (1): histologically confirmed prostate adenocarcinoma and with no more than 5 oligometastases, excluding regional lymph nodes by imaging examinations (2), no previous radiotherapy or RP for omPCa, and (3) treatment of primary tumor sites and metastatic lesions both with radiotherapy undergone at our institution. As the study cohort included both hormone-sensitive and castration-resistant prostate cancer (CRPC) patients, a subgroup analysis was planned to evaluate treatment efficacy within these different disease states. The majority of patients received ADT as the standard of care (SOC). In this study, omPCa was defined as the presence of five or fewer metastatic lesions, including involvement of extra-pelvic lymph nodes and bone metastases.

All patients underwent 68-Ga PSMA PET/CT, 68-Ga prostate-specific membrane antigen positron emission tomography-magnetic resonance (PSMA PET/MR), whole-body magnetic resonance imaging (WB-MRI), and/or single-photon emission computed tomography (SPECT) for staging prior to radiotherapy treatment. In cases where there was uncertainty, magnetic resonance imaging (MRI) was used to confirm the stage of the disease. PSA is the most crucial biomarker in the management of prostate cancer, as it represents the primary indicator of disease control. Routine monitoring of serum PSA and testosterone level is critical for evaluating the efficacy of treatment and detecting any potential disease recurrence. Hence, we conduct PSA regular monitoring at the time of diagnosis, prior to radiation therapy, one month after radiation therapy and monthly thereafter.

### Delivery of radiation

CT scans with a slice thickness of 5 mm of conventional radiotherapy or 1.5mm of SBRT and a scanning range of at least 10 cm above and below the prostate were used to identify the target area. The primary tumor’s clinical target volume (CTV) included the entire prostate and seminal vesicles (18). The planning target volume (PTV) was established to account for the tumor’s movement and was larger than the CTV by 0.5 cm in all directions.

For the treatment of the primary tumor, 32 patients received volumetric modulated arc therapy (VMAT), which was administered daily from Monday to Friday. The seminal vesicles received a prescribed dose of 68 (range 39.6-76) Gy, and the prostate received 70 (range 36.9-76) Gy, both delivered in 2 (range, 1.8-3) Gy per fraction. Three patients received moderate hypofractionation: two received a dose of 60 Gy in 20 fractions, while one received 70 Gy in 28 fractions. One patient received SBRT treatment with CyberKnife for the prostate and seminal vesicles, with a total dose of 37.5 Gy administered in five fractions every other day over a two-week period. The median equivalent dose in 2 Gy per fraction (EQD2) was approximately 70 (37.3-96.4) Gy, and the corresponding biologic equivalent dose (α/β=1.5Gy) (BED_1.5_) was 163.3 (87.1-225) Gy. The total radiation dose was tailored to the size and specific features of the lesions, as well as the volume of the tissue being treated. This was done to optimize the therapeutic outcome while keeping toxicity at an acceptable level.

Sixteen patients with 24 bone metastases and non-regional lymph nodes were treated with SBRT at a total dose of 27.5 to 37.1 Gy, which typically required 5 (range: 4-7) fractions for favorable outcomes. The BED_1.5_ ranged from 128.3 Gy to 211.5 Gy, with the median of 170.8 Gy. In contrast, 21 patients with bone metastases designated for conventional radiotherapy were treated with either VMAT or intensity-modulated radiotherapy (IMRT), with the technique selected based on lesion size and location. These patients typically received a dose of 45 Gy (range: 24–65 Gy) administered over 25 fractions (range: 8–33 fractions), resulting in a median BED_1.5_ of 99 Gy (range: 48–180 Gy). The relevant dosimetric parameters were summarized in **Table 1**.

**Table 1:** Treatment parameters used for radiotherapy.

Characteristic	Prostate primary lesion (fractionated radiotherapy)	Prostate primary lesion (SBRT)	Metastases (fractionated radiotherapy)	Metastases (SBRT)
Total prescribed dose (Gy)	70 (36.9-76)	37.5	45 (24–65)	32.5 (27.5-37.1)
Number of fractions	35 (20–38)	5	25 (8–33)	5 (4–7)
Dose per fraction (Gy)	2 (1.8-3)	7.5	2 (1.6-3)	6.4 (5.3-8.1)
BED_1.5_ (Gy)	163.3 (87.1-223.1)	225	42.4 (28.8-77.1)	73.2 (55-90.6)
EQD2 (Gy)	70 (37.3-95.6)	96.4	99 (48-180)	170.8 (128.3-211.5)

All data were shown as median values (range). BED_1.5_, biologic equivalent dose (α/β=1.5 Gy); EQD, equivalent dose in 2 Gy per fraction

### Response evaluation and follow-up

Upon completion of radiotherapy, monitoring of PSA levels was crucial for patients with prostate cancer as it provided valuable insights into treatment response and guided clinical decision-making. Therefore, patients underwent monthly PSA level checks to assess their disease status and identify any indications of disease recurrence. Biochemical progression was defined as a PSA increase of ≥ 2 ng/mL above nadir after radiotherapy (19). Overall survival (OS) was defined as the time interval from the initiation of radiotherapy to the last follow-up or the time of patient mortality. Biochemical progression-free survival (bPFS) was defined as the time interval from the start of radiotherapy until biochemical progression, or until the last follow-up for patients without progression. rPFS was defined as the time interval from the initiation of radiation therapy until radiographic evidence of disease progression or patient death, or until the last follow-up. Acute and chronic toxicities following radiation therapy were assessed using the Common Terminology Criteria for Adverse Events (CTCAE) v5.0.

### Statistical analysis

The rates of OS, rPFS, and bPFS were determined using the Kaplan-Meier method, and potential factors associated with these indicators were identified through univariate and multivariate Cox proportional hazard regression models. Variables with a p-value of ≤ 0.1 in the univariate analysis were included in the multivariate analysis. The final model for OS included a history of transurethral resection of the prostate (TURP) prior to EBRT, symptomatic status, and CRPC status. For rPFS, the model included the pre-radiotherapy PSA level, use of systemic therapy, and CRPC status. In the case of bPFS, the covariates were the BED_1.5_, Gleason Score, and site of metastases. Statistical analysis was performed using SPSS version 26.0 (IBM Corporation, Armonk, NY, USA). A p-value of less than 0.05 was considered statistically significant.

## Results

### Patient characteristics

This study analyzed 33 patients, with a median age of 70 years (range 51-90) at the time of diagnosis and treatment. The median PSA level was 76 ng/mL at the time of initial diagnosis, while it was 13.221 ng/mL before radiotherapy. The PSA level decreased significantly to 0.184 ng/mL (range 0.009-17.792) one month after radiotherapy. Before radiotherapy, only two patients had been diagnosed with CRPC. The majority of patients, 30 out of 33 (90.1%), including two CRPC patients, received concurrent ADT, with one patient receiving flutamide, two receiving apalutamide, and four receiving abiraterone. The median ADT duration was 43.1 months. Chemotherapy was administered to only one of the patients. Thirteen patients underwent transurethral resection of the prostate (TURP) prior to radiotherapy to alleviate urinary symptoms/obstruction. Among 33 patients, 13 (39.4%) had only one distant metastatic lesion, while 12 patients had two distant metastatic lesions. The remaining patients had three or more distant metastatic lesions. 23 patients (73.9%) were asymptomatic, while 10 patients (26.1%) presented symptoms related to the primary tumor or metastases. Detailed information on patient characteristics is shown in **Table 2**.

**Table 2:** Patients demography and tumor characteristics.

Characteristics	Values	Characteristics	Values
Age -years	70 (51-90)	Number of metastases/patient	
▪ ≦ 70	19/33 (57.6%)	▪ 1	13/33 (39.4%)
▪ > 70	14/33 (42.4%)	▪ 2	12/33 (36.4%)
Age at treatment time-years	70 (51 -90)	▪ 3	5/33 (15.2%)
Gleason Score		▪ 4	2/33 (6.1%)
▪ Grade1 (3 + 3)	2/33 (6.0%)	▪ 5	1/33 (3.0%)
▪ Grade2 (3 + 4)	2/33 (6.0%)	Systemic therapy	
▪ Grade3 (4 + 3)	5/33 (15.2%)	▪ Chemotherapy	1/33 (3.0%)
▪ Grade4 (4 + 4,3 + 5,5 + 3)	10/33 (30.3%)	▪ Androgen deprivation therapy	30/33 (90.1%)
▪ Grade5 (4 + 5,5 + 4,5 + 5)	14/33 (42.4%)	Site of oligometastatic disease	
PSA level at diagnosis-ng/ml	76 (8.4–9999)	▪ Bone metastases	33 (100%)
PSA level pre-EBRT-ng/ml	13.221 (0.040–187.4)	▪ Bone and non-regional nodal metastases	3 (39.1%)
PSA level 1 month after EBRT-ng/ml	0.184 (0.009–17.8)	SBRT involvement	
Symptoms		▪ Yes	17 (51.5%)
▪ Presented	10/33 (30.3%)	▪ None	16 (48.5%)
▪ None	23/33 (69.7%)	Castration-sensitivity before EBRT	
		▪ mHSPC	31/33 (93.9%)
		▪ mCRPC	2/33 (6.1%)

PSA, Prostate specific antigen; SBRT, Stereotactic body radiation therapy; EBRT, External beam radiation therapy; mHSPC, Metastatic hormone-sensitive prostate cancer; mCRPC, Metastatic castration-resistant prostate cancer.

### Efficacy outcomes

**Figure 1:**
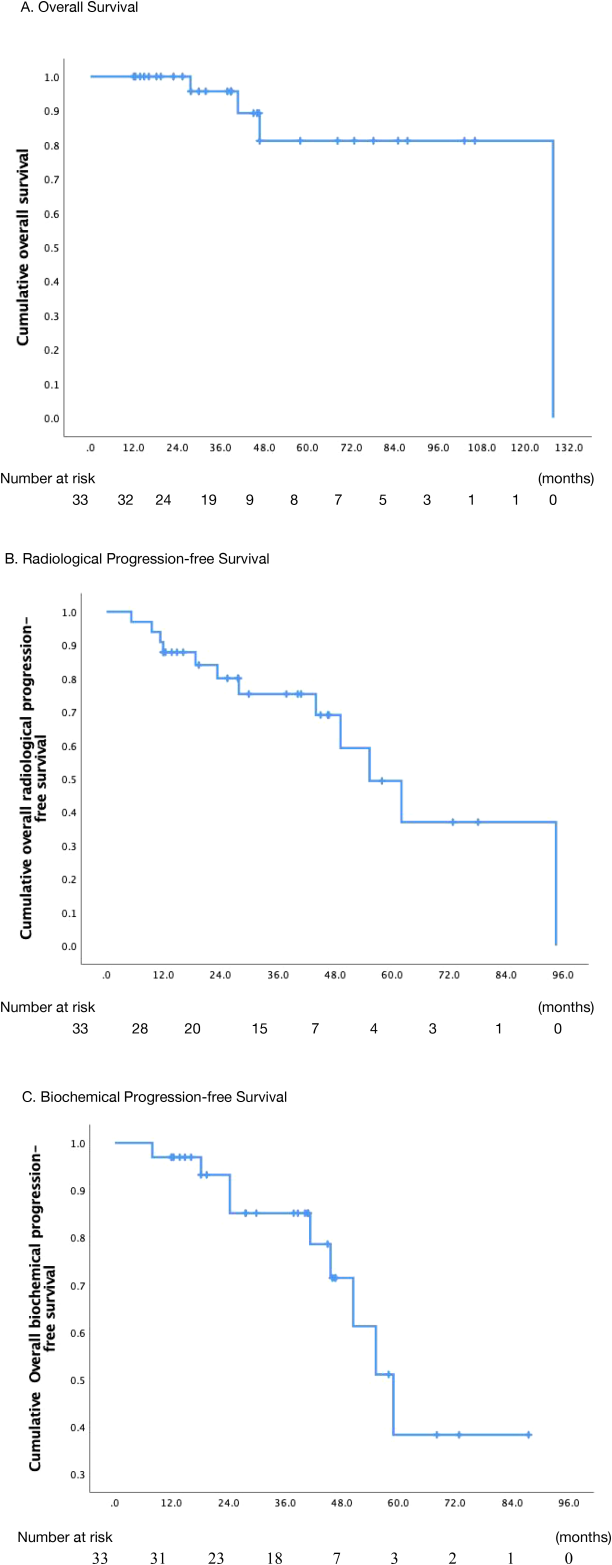
Actuarial survival analysis of patients. **(A)** Overall survival. **(B)** Overall radiological progression-free survival. **(C)** Overall biochemical progression-free survival. .

The median follow-up was 38.8 months (range: 4.2–70.6 months). The OS rates of 2-, 3-, and 5-year after treatment were 100.0%, 95.7%, and 81.2%, respectively (**Figure 1A**). Ten patients presented with symptoms before radiotherapy, such as urinary frequency and urgency, as well as local pain and other symptoms of metastatic lesions. After treatment, all patients experienced varying degrees of relief.

At the last follow-up, 4 patients (12.1%) died while 29 (87.9%) were alive. One patient died of distant metastases, two patients experienced local progression during radiotherapy, and one patient died due to non-tumor-related reasons. In the univariate analysis, for patients who underwent TURP before radiotherapy, the 5-year OS rate was 59.3%, while for patients without TURP before radiotherapy, the 5-year OS rate was 100% (p=0.04). Similarly, patients who presented oligometastatic or primary lesion-induced symptoms prior to the treatment had a 5-year OS of 68.2%, while those without symptoms had a 5-year OS of 100% (p=0.003). Moreover, the pre-radiotherapy status of CRPC decreased the OS rate of patients significantly (p=0.006). Although PSA played a significant role in the diagnosis, treatment, and evaluation of efficacy, we did not find any correlation between PSA and OS (PSA pre-EBRT-ng/ml ≤ 3 *VS*.>3, p=0.135). However, in the univariate analysis, none of the aforementioned predictors were significantly associated with OS. Results were summarized in **Table 3**.

**Table 3:** Univariate analysis for OS rate.

Characteristic	2-year OS rate (%)	3-year OS rate (%)	5-year OS rate (%)	P value
Age-years				0.854
≦70	100	92.9	82.5	
>70	100	100	75.0	
Gleason Score				0.141
≦8	100	93.3	93.3	
> 8	100	100	40.0	
PSA at diagnosis-ng/ml				0.273
≦20	100	100	100	
>20	100	94.4	73.6	
PSA pre-EBRT-ng/ml				0.135
≦3	100	100	100	
>3	100	93.8	66.7	
PSA post-EBRT-ng/ml				0.296
≦1	100	94.1	74.9	
>1	100	100	100	
Number of metastases				0.288
1	100	100	62.5	
>1	100	93.3	93.3	
Site of metastases				0.641
Bone	100	95.5	79.8	
Bone and non-regional nodal	100	100	100	
TURP before EBRT				0.040
Yes	100	88.9	59.3	
None	100	100	100	
Systemic therapy (ADT or Chemotherapy)				0.571
Yes	100	95.0	79.4	
None	100	100	–	
Symptoms				0.003
Presented	100	90.9	68.2	
None	100	100	100	
SBRT involvement				0.263
Yes	100	100	87.5	
No	100	90.9	72.7	
CRPC				0.006
No	100	95.5	86.8	
Yes	100	100	0	

OS, Overall survival; TURP, Transurethral resection of the prostate; EBRT, External beam radiation therapy; SBRT, Stereotactic body radiation therapy;CRPC, Castration Resistant Prostate Cancer.

The median rPFS was 55.3 months (95% CI: 39.9 to 70.8 months), with the corresponding rates at 2-, 3-, and 5-year rPFS rate of 80.1%, 75.3%, and 49.3%, respectively (**Figure 1B**). In the univariate analysis, it was observed that the 3-year rPFS rate was 0% for two patients who were diagnosed with CRPC before radiotherapy, while it was 80.2% for the remaining patients (p<0.001). Moreover, combination with ADT or chemotherapy showed a trend toward improved rPFS, but the correlation was not statistically significant (p=0.063). Patients with a pre-radiotherapy PSA level of ≤ 1.0 ng/ml appeared to have a higher likelihood of disease recurrence or progression (p=0.056); however, this counterintuitive finding should be interpreted with extreme caution, given the small sample size and potential confounders inherent to this retrospective series. In the multivariate analysis of rPFS rates, with combination ADT (HR = 9.871, 95%CI (1.540-63.263), p= 0.016), metastatic hormone-sensitive prostate (mHSPC) (HR = 52.555, 95% CI (5.776-478.226), p<0.001), and PSA levels > 1 ng/ml before radiation therapy (HR = 7.596, 95% CI (1.498-38.192), p= 0.014) were independent prognostic factors (table sup1). The relevant information was indicated in **Table 4**.

**Table 4:** Univariate analysis for rPFS rate.

Characteristic	2-year rPFS rate (%)	3-year rPFS rate (%)	5-year rPFS rate (%)	P value
Age-years				0.368
≦70	70.7	70.7	42.4	
>70	92.9	81.3	65.0	
Gleason Score				0.143
≦8	85.0	85.0	60.7	
>8	72.7	60.6	40.4	
PSA pre-EBRT-ng/ml				0.056
≦1	37.5	37.5	0	
>1	85.4	80.0	60.6	
PSA at diagnosis-ng/ml				0.927
≦20	68.6	68.6	45.7	
>20	83.3	76.9	46.1	
PSA post-EBRT-ng/ml				0.919
≦1	82.5	76.2	45.7	
>1	75.0	75.0	56.3	
Number of metastases				0.713
1	75.0	75.0	75.0	
>1	83.7	77.2	40.5	
Site of metastases				0.103
Bone	82.3	77.2	55.1	
Bone and non-regional nodal	66.7	66.7	0	
Systemic therapy (ADT or Chemotherapy)				0.063
Yes	81.2	76.1	54.4	
No	66.7	66.7	0	
TURP before EBRT				0.441
Yes	66.6	66.6	50.0	
No	90.0	81.8	46.8	
Symptoms				0.512
Presented	70.7	70.7	–	
None	91.7	83.3	52.9	
CRPC				≤0.001
No	85.2	80.2	52.5	
Yes	0	0	0	
SBRT involvement				0.107
Yes	75.5	67.9	35.7	
No	85.2	85.2	85.2	

rPFS, Radiological progression-free survival; PSA, Prostate-specific antigen; ADT, Androgen deprivation therapy; TURP, Transurethral resection of the prostate; EBRT, External beam radiation therapy; CRPC, Castration resistant prostate cancer;SBRT, Stereotactic body radiation therapy.

The median bPFS was 58.9 months (95% CI, 47.7 - 70.1 months), and the bPFS rates at 2-, 3-, and 5-year were 93.2%, 85.1%, and 38.3%, respectively (**Figure 1C**). Univariate analysis revealed that patients with both non-regional lymph node and bone metastases had a 3-year bPFS of 66.7%, while those with only bone metastases had a 3-year bPFS of 87.9% (p=0.005). Multivariate analysis confirmed that patients with both non-regional lymph node and bone metastases had a higher likelihood of biochemical recurrence (HR = 8.823, 95% CI (1.419-54.848), p=0.02). In univariate analysis, although patients receiving higher BED_1.5_ (>170 Gy *VS*. ≤170 Gy) may have better biochemical control, the relationship was not significant (p=0.078). The specific details were described in **Table 5**.

**Table 5:** Univariate analysis for bPFS rate.

Characteristic	2-year bPFS rate (%)	3-year bPFS rate (%)	5-year bPFS rate (%)	P value
Age-years				0.841
≦70	93.8	80.4	41.3	
>70	92.9	92.9	0	
BED_1.5_-Gy				0.078
≧160	100	100	40.0	
<160	83.9	65.3	43.5	
Gleason Score				0.071
≦8	100	93.3	44.4	
>8	83.1	72.7	54.5	
PSA pre-EBRT- ng/ml				0.128
≦3	88.9	63.5	19.0	
>3	94.7	94.7	69.1	
PSA at diagnosis- ng/ml				0.918
≦20	83.3	83.3	41.7	
>20	96.2	85.5	37.4	
PSA post-EBRT- ng/ml				0.296
≦1	100	94.1	85.6	
>1	100	100	100	
Number of metastases				0.962
1	91.7	80.2	40.1	
>1	94.4	88.1	41.1	
Site of metastases				0.005
Bone	96.7	87.9	42.8	
Bone and non-regional nodal	66.7	66.7	0	
Systemic treatment(ADT/Chemotherapy)				0.507
Yes	92.5	83.2	40.5	
No	100	100	0	
TURP before EBRT				0.883
Yes	91.7	81.5	30.6	
No	95.0	88.2	42.9	
Symptoms				0.687
Presented	88.4	80.4	–	
None	100	91.7	39.3	
CRPC				0.622
No	92.7	84.3	37.9	
Yes	–	–	–	
SBRT involvement				0.297
Yes	93.3	93.3	42.0	
No	93.8	76.7	51.1	

bRFS, Biochemical progression-free survival; BED1.5, Biologically effective dose (α/β=1.5Gy); PSA, Prostate-specific antigen; ADT, Androgen deprivation therapy; TURP, Transurethral resection of the prostate; EBRT, External beam radiation therapy; CRPC, Castration resistant prostate cancer;SBRT, Stereotactic body radiation therapy.

### Treatment toxicity

Thirteen patients in total experienced acute reactions, with eight of them having genitourinary (GU) toxicity. The majority of patients (7/8) had grade 1 toxicity reactions, and all of them spontaneously recovered after undergoing radiotherapy. Ten patients experienced acute gastrointestinal (GI) toxicity, with nine of them having mild diarrhea. There were no acute adverse reactions greater than grade 2. One patient developed intestinal bleeding after radiotherapy and was subsequently diagnosed with chronic radiation enteritis after undergoing further colonoscopy. There were no patients with chronic GU toxicity in the study. Out of all patients, only one experienced chronic Grade 3 gastrointestinal toxicity, resulting in a toxicity rate of 3.03%. No Grade 4 or higher adverse events were observed.

## Discussion

Currently, systemic therapy is the main treatment for oligometastatic prostate cancer. Literature evidence supports local primary treatment in this setting. On the other hand, adding metastases directed therapy to local primary treatment remains under investigation in this context. The present study aimed to investigate the efficacy of radiotherapy for both primary and metastatic lesions in omPCa.

Regarding the novel endocrine therapy, N.D. James et al. evaluated the use of abiraterone acetate and prednisolone in treating prostate cancer patients based on a multi-arm, multi-stage trial. Their results showed a 3-year failure-free survival rate of 75% and a median failure-free survival of 43.9 months (20). A study on abiraterone in patients with mHSPC has demonstrated a median bPFS of 33.2 months and a median rPFS of 33.0 months (8). In the randomized, double-blind, phase III trial (LATITUDE) evaluating the treatment of abiraterone acetate and prednisone in newly diagnosed high-risk mHSPC patients, the overall survival period of the abiraterone acetate and prednisone combined with ADT group (median 53.3 months; 95% CI 48.2 - not reached) was significantly longer than the placebo plus ADT group (36.5 months; 95% CI 33.5 - 40.0). The median PFS was also longer in the abiraterone acetate and prednisone group (33.3 months) compared to the placebo group (7.4 months) (21). Our retrospective study included oligometastatic patients who received radiotherapy for both primary and metastatic lesions without definitive surgical treatment. The median follow-up was 38.8 months. The 2-year and 5-year OS rates were 100.0% and 81.2%, respectively. The median rPFS and bPFS were 55.3 (95% CI 39.8-70.8) months and 58.9 (95% CI 47.1-70.1) months, respectively, and the 3-year rPFS and bPFS rates were 75.3% and 85.1%, respectively.

Y. Cho conducted a case-control study that enrolled a total of 140 patients with metastatic prostate cancer. Of these patients, 38 received radiation therapy at the primary prostate site, 39 received palliative radiation therapy, and 63 did not receive any radiation therapy. The results showed that the group that received radiation therapy to the primary prostate site had a higher 3-year OS rate than patients without local treatment (69% *vs*. 43%, p=0.004). Additionally, there was a benefit in terms of 3-year bPFS rate (52% *vs*. 16%, HR = 0.43, p=0.015) (22). A review article on treatment options for omHSPC suggests that primary local therapy may improve the prognosis of low-volume metastatic disease (23).The STAMPEDE trial showed that in low-burden metastatic prostate cancer patients, those who received hormone therapy and prostate radiotherapy had improved OS compared to the control group (hormone therapy or hormone therapy plus docetaxel) (HR 0.68, 95% CI 0.52-0.90; p=0.007). The 3-year survival rate was 81% for patients receiving radiotherapy and 73% for those receiving systemic treatment. While in the subgroup with high volume, there was no improvement in either the failure-free survival rate or the OS rate (15). The survival benefit of prostate-directed radiotherapy was further solidified by the STOPCAP individual patient data meta-analysis, which synthesized data from the STAMPEDE and HORRAD trials. STOPCAP not only confirmed the OS benefit (HR 0.68) in patients with low metastatic burden but also provided a more robust quantitative definition, suggesting the benefit is most pronounced in patients with fewer than five bone metastases. By establishing that the benefit is confined to this low-burden subgroup, STOPCAP provides a strong rationale for investigating even more aggressive, comprehensive treatment strategies, such as the one employed in our study, where all sites of disease were targeted (14). The PEACE-1 trial offers the latest high-level evidence on the utility of prostate radiotherapy administered concurrently with an intensified, modern systemic regimen (ADT, docetaxel, and abiraterone). Combining radiotherapy with standard of care plus abiraterone improved rPFS and castration resistance-free survival, but not overall survival in patients with low-volume *de novo* metastatic castration-sensitive prostate cancer. Radiotherapy reduced the occurrence of serious genitourinary events, regardless of metastatic burden and without increasing the overall toxicity.

A systematic research analysis has demonstrated the effectiveness of prostate-directed therapy (PDT) combined system treatment in patients with metastases to non-regional lymph nodes (M1a stage) (25). Local treatment for the primary lesion can serve the purpose of reducing the tumor burden, alleviating symptoms, and providing survival benefits. The underlying principle was that the primary tumor was the origin of metastatic cancer cells, and proactive management of the primary tumor can impede the advancement of metastatic lesions as well as the development of new metastases (25). The addition of prostate radiation therapy to SOC treatments can significantly improve the 3-year failure-free survival rate (51% *vs*. 29%, HR 0.63, 95% CI 0.42-0.94) in *de novo* M1a PCa patients (24, 26). Our study showed a 3-year survival rate of 95.7%. The median rPFS was 55.3 months (95% CI 39.8 to 70.8), and the 3-year rPFS rate was 75.3%. These favorable results may be due to our approach of administering local treatment for the primary site and metastatic burden at the same time. This may be due to our approach of administering local treatment for the primary site and metastatic burden at the same time using either conventional fractionation radiotherapy or SBRT to control all clinical tumor burden. In our study, both IMRT and VMAT were used, yielding excellent survival outcomes consistent with existing literature on their therapeutic equivalence. Our findings support using either technique as an effective part of a comprehensive radiotherapy strategy for oligometastatic disease.

Patients with omPCa may benefit from a combination of treatment methods, including LCT and systemic therapy. C. Reverberi et al. conducted a study similar to ours, focusing on LCT for the primary site and metastatic tumor burden in newly diagnosed omPCa patients (27). The difference was that they also analyzed patients who underwent radical surgery for the primary lesion, with a median bPFS of 58 months and 2-year and 5-year bPFS rates of 73.3% and 39.3%, respectively. Their study showed 2-year and 5-year local recurrence-free survival rates of 93.9% and 83.7%, respectively. The bPFS was comparable to our study, while the rPFS was slightly better. This difference may be attributed to the fact that their study included patients who had undergone radical prostatectomy, whereas in our study, some patients received palliative radiotherapy with a lower radiation dose. Their study found a significant correlation that patients with post-treatment PSA levels ≤ 1 ng/mL had high bPFS rates (p = 0.004). In contrast, our study revealed that patients with pre-treatment PSA levels below 1 ng/ml due to the application of ADT before radiotherapy were more likely to experience disease progression (p=0.014). This might be attributed to the nature of the retrospective study and the limited patients enrolled.

In our univariate analysis of bPFS, there was a non-significant trend toward improved biochemical control in patients receiving BED_1.5_ ≥ 170 Gy (p = 0.078). While conventional EBRT guidelines recommend 78 Gy/39 f, and SBRT protocols typically prescribe 42.7 Gy/7 f or 36.25 Gy/5 f for definitive intent (4), our SBRT cohort met these benchmarks and showed OS comparable to the overall study population. Given the oligometastatic nature of our patient cohort, treatments were primarily palliative in dose. We propose that escalating the radiation dose to the primary lesion—within patient-tolerance limits—may enhance lesion control. Although our dosimetric variables did not reach statistical significance, this hypothesis warrants further investigation to optimize outcomes.

In the evolving landscape of treating oligometastatic prostate cancer with comprehensive radiotherapy, several other studies are worth mentioning. Imber et al. evaluated 47 *de novo* oligometastatic patients treated with combined prostate- and metastases-directed RT, reporting clinical outcomes with a median follow-up of 27 months (28). Other series have explored this approach in smaller cohorts, such as Aizawa et al., who included 16 patients receiving ≥ 70 Gy via EBRT (29), and Inaba et al., who assessed 35 patients with extended follow-up (30). While much of the existing data is retrospective, maturing prospective evidence is emerging from trials such as the SOLAR Phase 2 study (31). The therapeutic strategies have also been diverse: Deantoni et al. investigated patients with low-burden bone metastases (32), whereas Tsumura et al. examined the role of prostate brachytherapy combined with metastases-directed RT (33). In contrast to these varied approaches, our study exclusively utilized EBRT for all sites, with the unique application of CyberKnife SBRT to metastatic lesions in a subset of patients.

The occurrence of acute adverse reactions of Grade 2 or higher was not observed, while the incidence of chronic adverse reactions of Grade 3 was 3.03%. The main adverse events observed in this study were mild GU and GI toxicity, with only one patient experiencing grade 3 chronic gastrointestinal toxicity, indicating that this treatment was well-tolerated by patients. The patient presented symptoms of bleeding during a colonoscopy.

This study’s primary limitations stem from its retrospective, single-center design, which introduces potential selection bias and treatment heterogeneity (e.g., disease setting, variations in radiation dosing and systemic therapies), which limit the generalizability of our findings and make it challenging to isolate the precise effect of the comprehensive radiotherapy strategy from confounding variables. Furthermore, the small sample size reduces statistical power, potentially masking more subtle treatment benefits. The median follow-up period was also insufficient to fully assess long-term outcomes, such as late recurrence and toxicity. Despite these constraints, this study provides valuable hypothesis-generating data. An exploratory analysis of tumors in patients with clinical oligometastatic disease and mixed histology who underwent SBRT at all known sites revealed the potential of miR-23b, miR-449a, and miR-449b as prognostic markers for predicting survival in 17 patients with available expression data (34). Future large-scale, prospective, multi-center trials are required to definitively validate these preliminary findings and further investigate the role of these biomarkers in patient stratification.

## Conclusion

Local radiation therapy on the primary tumor as part of *de novo* metastatic, hormone-sensitive prostate cancer is recommended for low-burden oligometastatic disease since 2018. Furthermore, studies on stereotactic body radiotherapy for oligometastases have shown that this therapeutic approach is feasible and effective. As one of the rare studies in the literature about definitive radiotherapy for local and metastatic lesions, the study showed it was a safe and effective treatment modality with favorable outcomes for omPCa. These findings suggest that definitive radiotherapy for the primary site and oligometastases may be a feasible and well-tolerated treatment option for patients with oligometastatic prostate cancer. Notably, a high level of evidence is still pending.

## Data Availability

The original contributions presented in the study are included in the article/**Supplementary Material**. Further inquiries can be directed to the corresponding authors.

## Glossary

omPCa: oligometastatic prostate cancer
OS: overall survival
bPFS: biochemical progression-free survival
rPFS: radiological progression-free survival
omHSPC: oligometastatic hormone-sensitive prostate cancer
omCRPC: oligometastatic castration-resistant prostate cancer
TURP: transurethral resection of the prostate
ADT: androgen deprivation therapy
CRPC: castration-resistant prostate cancer
PSA: prostate-specific antigen
PCa: prostate cancer
mHSPC: metastatic hormone-sensitive prostate cancer
ARSI: androgen receptor signaling inhibitor
mCRPC: metastatic castration-resistant prostate cancer
PSMA PET/CT: prostate-specific membrane antigen positron emission tomography-computed tomography
mPCa: metastatic prostate cancer
RP: radical prostatectomy
LCT: local consolidative treatment
QoL: quality of life
BRT: brachytherapy
SBRT: stereotactic body radiation therapy
EBRT: external beam radiation therapy
SOC: standard of care
PSMA PET/MR: prostate-specific membrane antigen positron emission tomography- magnetic resonance
WB-MRI: whole-body magnetic resonance imaging
SPECT: single-photon emission computed tomography
MRI: enhanced magnetic resonance imaging
CTV: clinical target volume
PTV: planning target volume
VMAT: volumetric modulated arc therapy
EQD2: equivalent dose in 2 Gy per fraction
BED: biologically effective dose
IMRT: intensity-modulated radiotherapy
CTCAE: Common Terminology Criteria for Adverse Events
GU: genitourinary
GI: gastrointestinal
PDT: prostate-directed therapy.
