# Supplementary material for: Definitive radiotherapy for local and metastatic lesions in prostate cancer patients with oligometastases
Source: Front Oncol. 2025 Nov 3;15:1662567. doi: 10.3389/fonc.2025.1662567 (PMC12620242; doi:10.3389/fonc.2025.1662567)
Supplement: Supplementary file 1 [file Table1.docx]

**Table sup.1. Multivariate analysis for rPFS rate**

| Variables | **r**PFS | | |
| --- | --- | --- | --- |
|  | HR | 95% CI | P value |
| PSA pre-EBRT-ng/ml |  |  |  |
| ≦1 vs. >1 | 7.596 | 1.498-38.192 | 0.014 |
| Systemic treatment(ADT/ Chemotherapy) |  |  |  |
| None vs.Yes | 9.871 | 1.540-63.263 | 0.016 |
| CRPC |  |  |  |
| Yes vs.No | 52.555 | 5.776-478.226 | 0.000 |

**rPFS: Radiological progression-free survival; EBRT: External beam radiation therapy; ADT: Androgen deprivation therapy; CRPC: Castration resistant prostate cancer.**
